# Supplementary material for: Rac1 Promotes Cell Motility by Controlling Cell Mechanics in Human Glioblastoma
Source: Cancers (Basel). 2020 Jun 23;12(6):1667. doi: 10.3390/cancers12061667 (PMC7352963; doi:10.3390/cancers12061667)
Supplement: Supplementary file 1 [file cancers-12-01667-s001.zip › cancers-826616.Supplementary materials.pdf]

Supplementary Materials

Rac1 Promotes Cell Motility by Controlling Cell Mechanics in Human Glioblastoma

Jing Xu, Nicola Galvanetto, Jihua Nie, Yili Yang and Vincent Torre

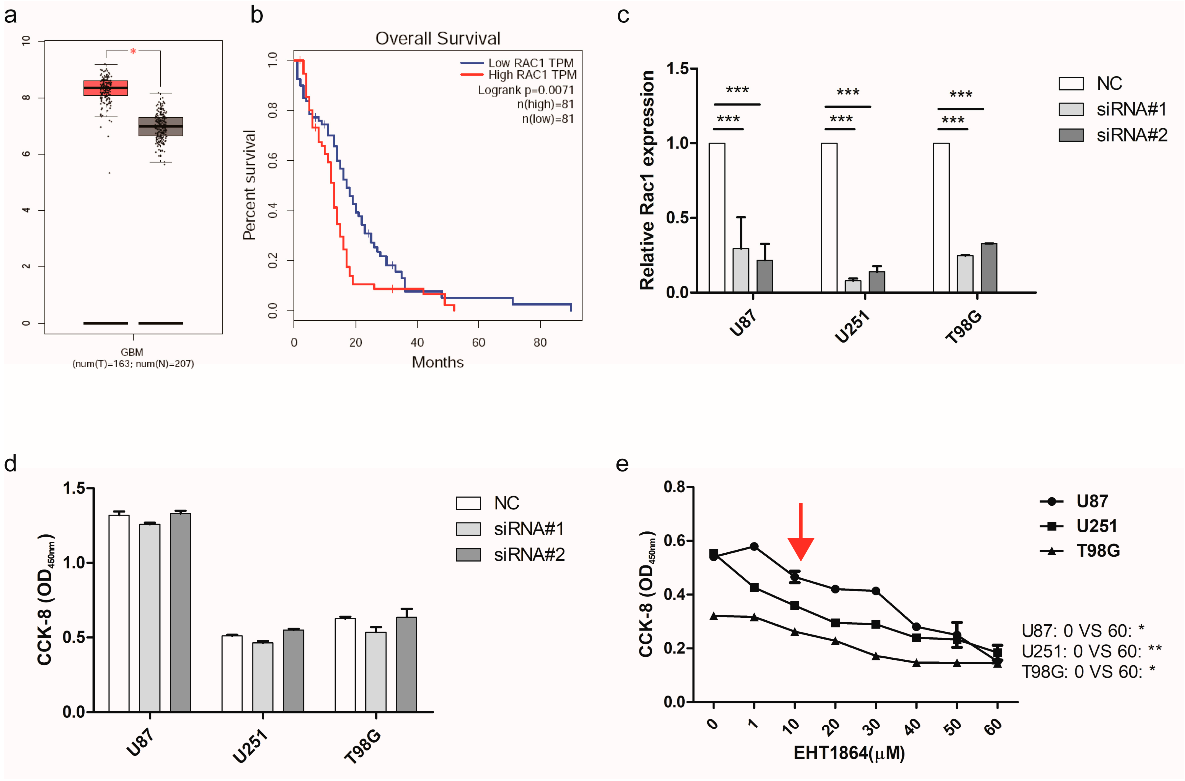

Figure S1. (**a**) Rac1 expression in GBM tissue compared with non-GBM tissue (The Y-axis represent log2 (TPM+1), red box represent GBM tissue and grey box represent non-GBM tissue). (Dataarefrom GEPIA: gepia.cancer-pku.cn) (**b**) Overall survival of GBM patients with low Rac1 expression and high Rac1 expression. (Data are from GEPIA: gepia.cancer-pku.cn) (**c**) mRNA levels of Rac1in U87, U251, and T98G GBM cells were analyzed by RT-PCR after Rac1-siRNA and NC-siRNA were transfected for 48h. (**d**)Cell viability of U87, U251, and T98G GBM cells was evaluated by using the CCK-8 assay after Rac1 knockdown for 48h. (**e**) Cell viability of U87, U251, and T98G GBM cells was evaluated by using the CCK-8 assay after incubation with different concentrations of EHT 1864 for 12 h. ***: p<0.001, **: p<0.01, *: p<0.05.

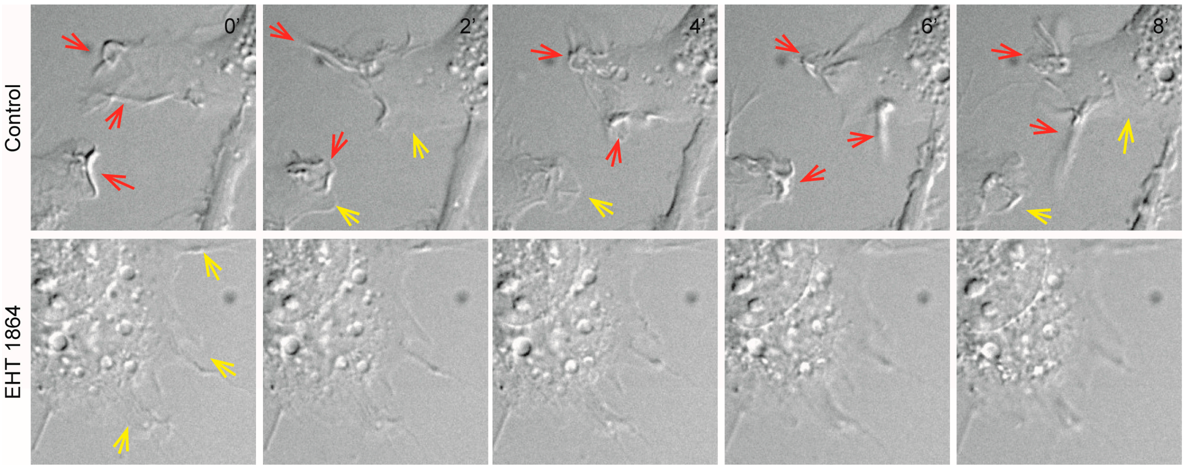

Figure S2. Phase contrast time-lapse microscopy recording of U87 cells at 2min intervals for 1h. After 1h recording, 10μm EHT 1864 was added and recorded for another 1h to observe cell membrane movement. The red arrow indicates cell membrane ruffles, and the yellow arrow indicates cell membrane protrusion. U87 cellrecordings are shown in Video S3.

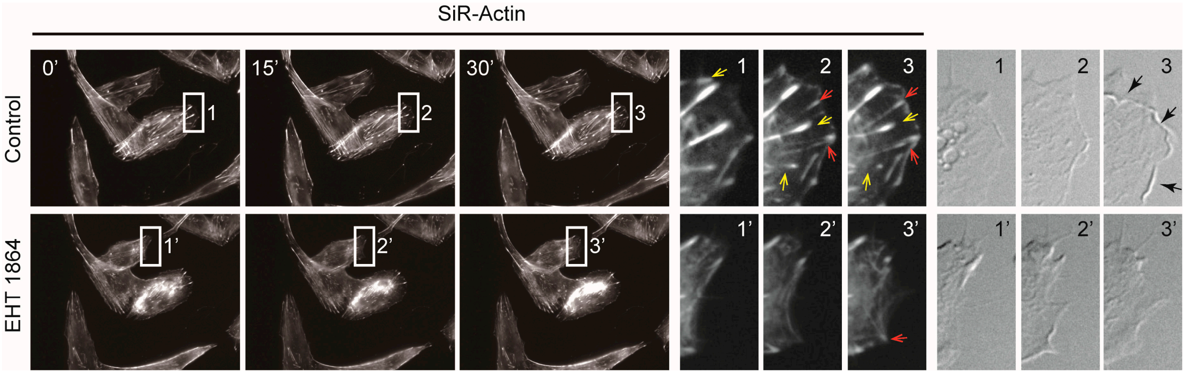

Figure S3. SiR-actin staining of U87 cells was imaged by time-lapse microscopy at 2min intervals for 30min. After 30min recording, 10μm EHT 1864 was added and recorded for another 30min to observe actin organization. The red arrow indicates actin assembly fibers in the protrusion, and the yellow arrow indicates actin disassembly fibers in the protrusion. Recordings of actin dynamics are shown in Videos S4 and S5.

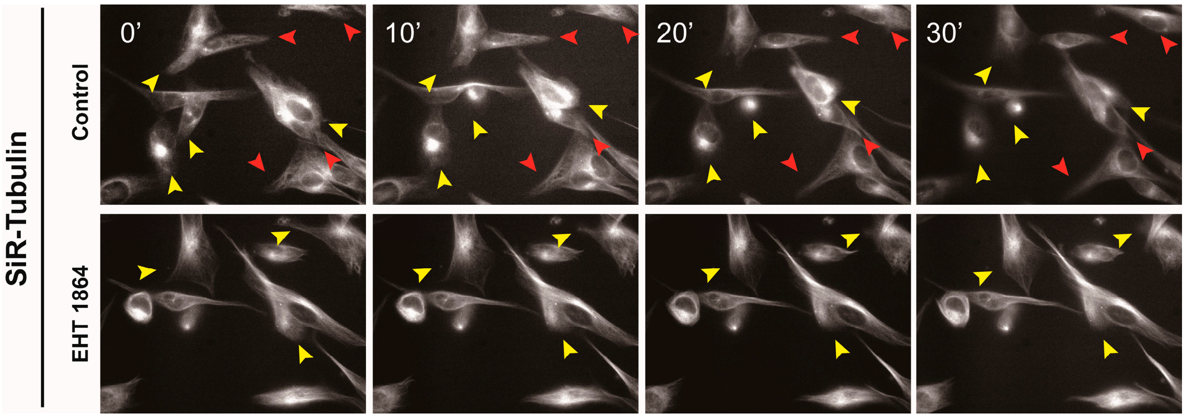

Figure S4. SiR-tubulin staining of U87 cells was imaged by time-lapse microscopy at 2min intervals for 30min. After 30min recording, 10μm EHT 1864 was added and recorded for another 30min to observe tubulin organization. The red arrow indicates tubulin assembly in the protrusion, and the yellow arrow indicates tubulin disassembly.

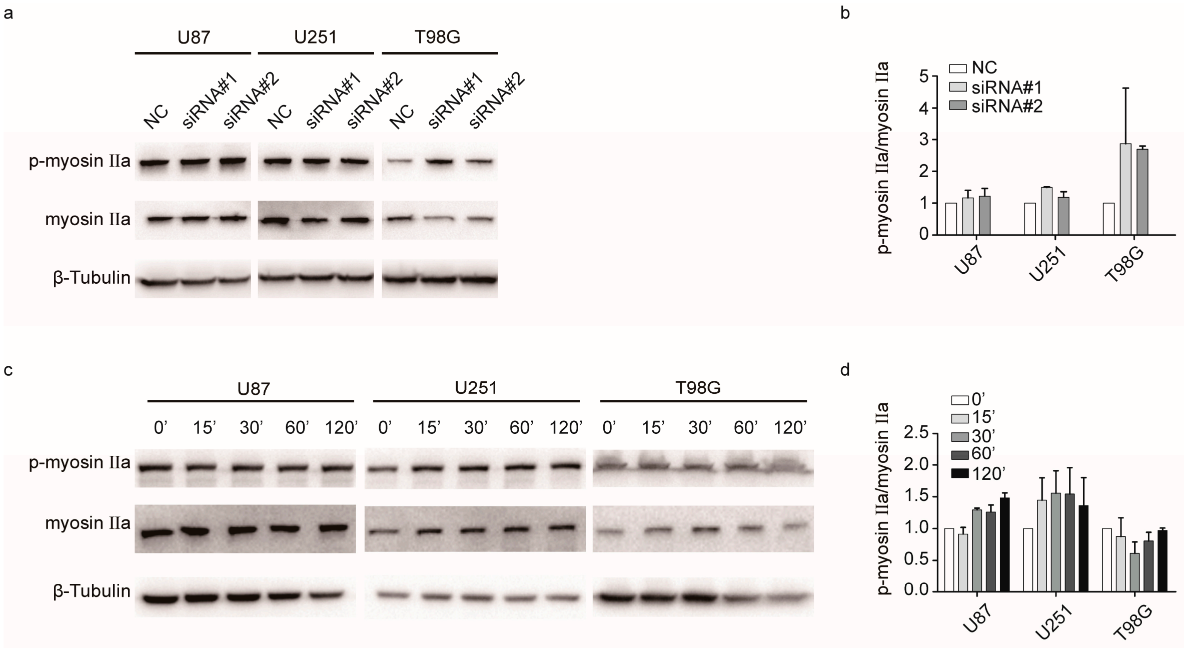

Figure S5. Western blot analysis of the level of phosphorylation of myosin IIa in U87, U251, and T98G cells in response to Rac1 depletion by siRNA and inhibited by EHT 1864.

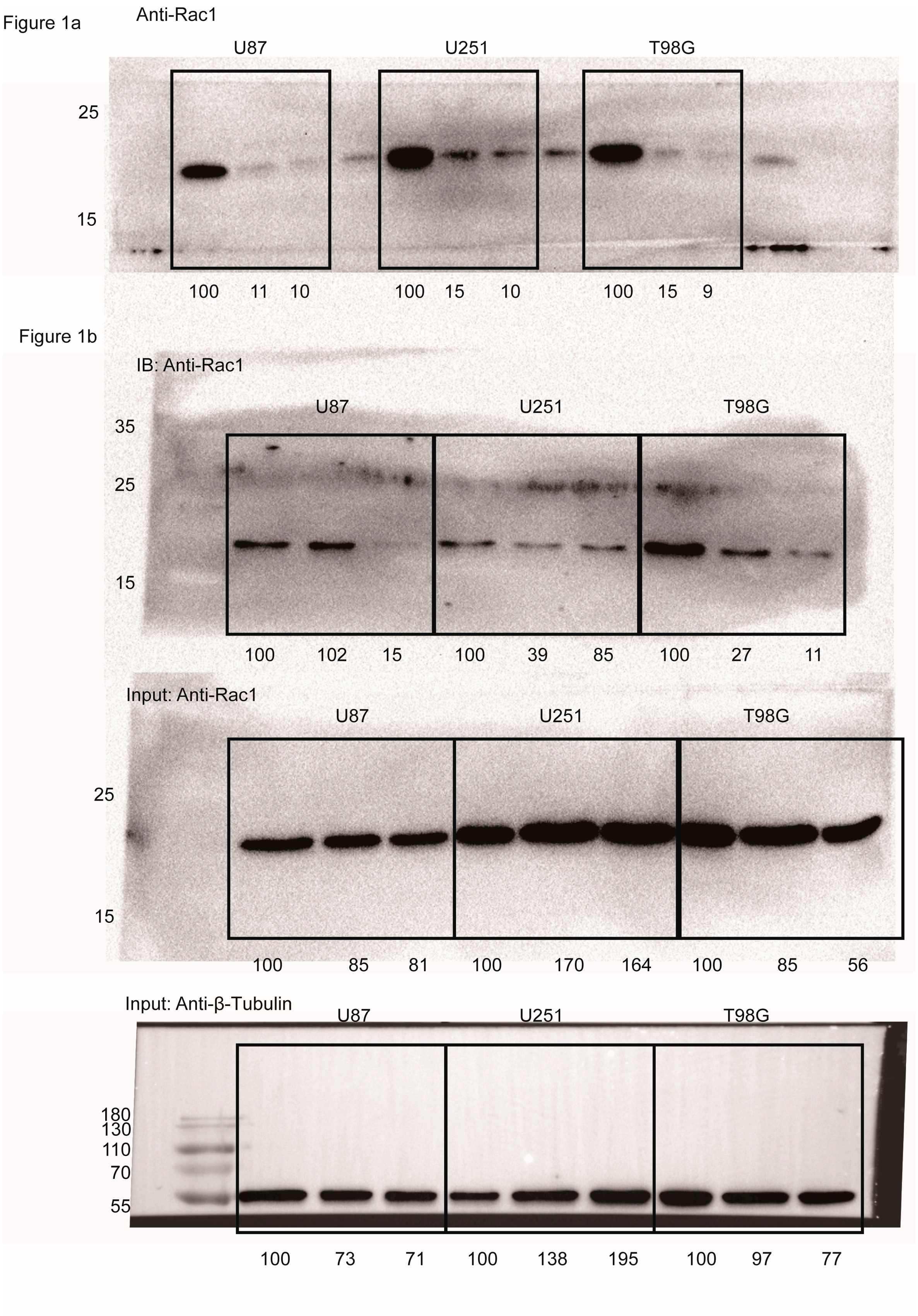

Figure S6. The whole Western Blot for Figure 1.

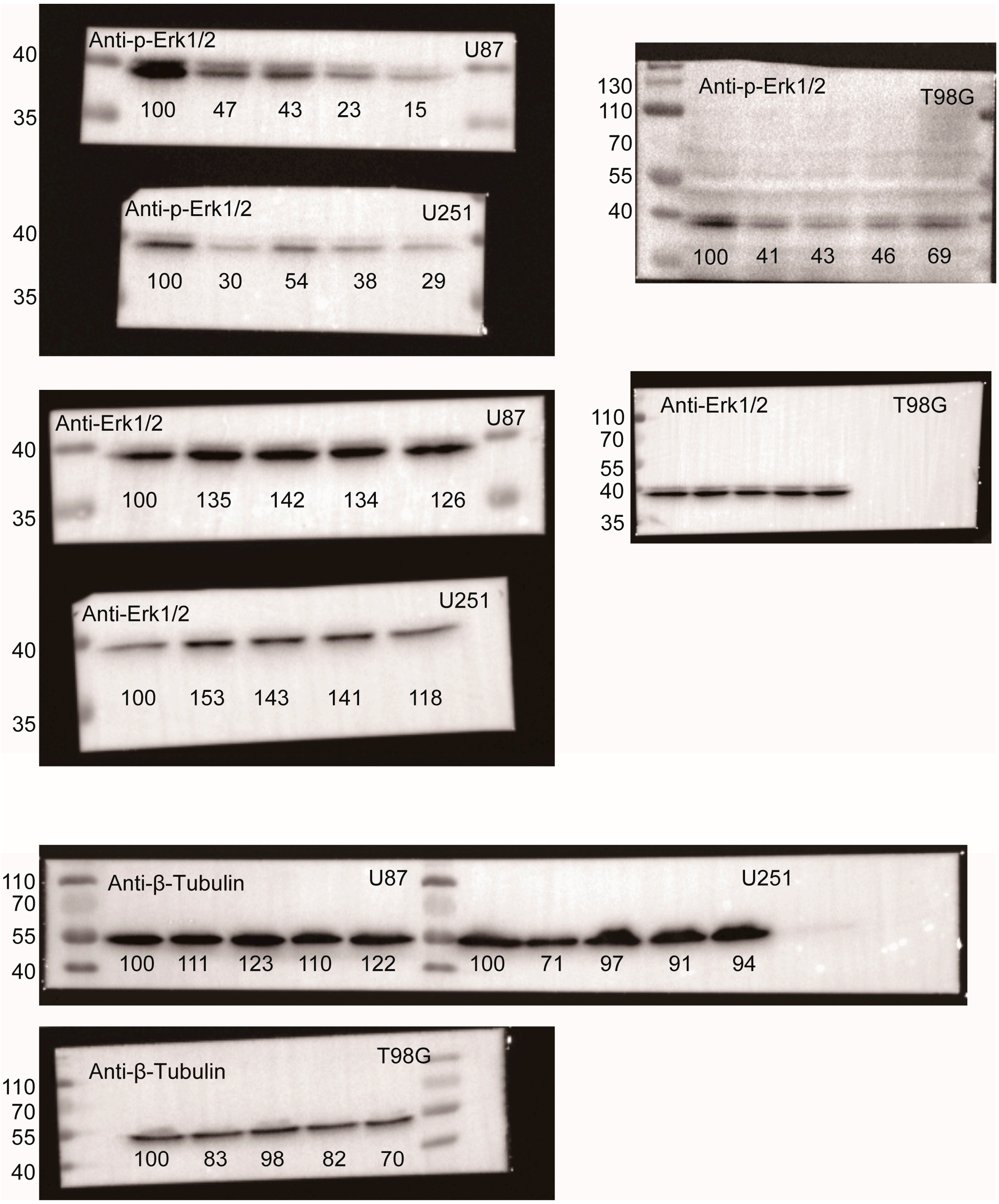

Figure S7. The whole Western Blot for Figure 6.

© 2020 by the authors. Licensee MDPI, Basel, Switzerland. This article is an open access article distributed under the terms and conditions of the Creative Commons Attribution (CC BY) license (http://creativecommons.org/licenses/by/4.0/).
